# Exploiting haem-iron dependence of nontypeable *Haemophilus influenzae*: an avenue for future therapeutic development

**DOI:** 10.3389/fcimb.2025.1548048

**Published:** 2025-05-15

**Authors:** Brianna Atto, David A. Gell, Robyn Marsh, Stephen Tristram

**Affiliations:** ^1^ School of Health Sciences, University of Tasmania, Launceston, TAS, Australia; ^2^ Menzies Institute for Medical Research, University of Tasmania, Hobart, TAS, Australia; ^3^ Child and Maternal Health Division, Menzies School of Health Research, Darwin, NT, Australia

**Keywords:** haem acquisition, new therapeutic agents, nontypeable *H. influenzae*, respiratory infections, iron, host-pathogen interactions, heme

## Abstract

Nontypeable *Haemophilus influenzae* (NTHi) is a major respiratory pathogen that imposes a substantial disease burden, globally. Further amplifying the burden of NTHi-associated infections is the rapidly expanding spectrum and prevalence of antibiotic resistance, and the lack of an effective vaccination strategy. In 2017, the World Health Organization list of “priority pathogens”, highlighted the urgent need for new therapeutic agents against NTHi. Consequently, alternative preventative or treatment approaches that do not rely on antibiotic susceptibility or stable vaccine targets are becoming more attractive. The nutritional dependency for haem/iron at all stages of NTHi pathogenesis exposes a vulnerability that may be exploited for the development of such therapies. This review explores the role of haem/iron in all facets of NTHi pathogenesis, the host-bacterial competition for this vital nutrient, and the therapeutic potential of strategies that interfere with its acquisition.

## 1 NTHi is a major pathogen in the respiratory tract

Members of the *Haemophilus* genus are Gram-negative coccobacilli belonging to the *Pasteurellaceae* family. Most infections associated with this genus are caused by *H. influenzae*; other species such as *H. aegyptius*, *H. parainfluenzae* and *H. ducreyi* are rarely isolated from clinical samples but have been documented to cause a variety of mild respiratory or genitourinary tract infections (Nørskov-Lauritsen, 2014; Van Eldere et al., 2014). *H. influenzae* can be subtyped into encapsulated strains, which express different serotypes of capsular polysaccharide (designated types a–f), and nonencapsulated strains, which are designated nontypeable *H. influenzae* or NTHi. Prior to widespread implementation of the *Haemophilus influenzae* type b (Hib)-conjugate vaccination programs in the 1990s, Hib was the most common cause of bacterial meningitis in children under the age of five (Heath et al., 2001; Maguire et al., 2018; Soeters et al., 2018). Concurrent with the decline in Hib disease, the prevalence of NTHi in human carriage and disease has increased such that NTHi is now the most common phenotype isolated from clinical infection sites (Van Eldere et al., 2014).

NTHi must first colonise the nasopharynx before migrating to other anatomical sites where it can cause a wide spectrum of disease; however, the exact mechanisms that influence the behavioural shift of NTHi from coloniser to pathogen are not understood (Van Eldere et al., 2014). Asymptomatic nasopharyngeal NTHi colonisation is common among adults (20-30%) and children under the age of five (52-84%) (Mackenzie et al., 2010; Bajanca-Lavado et al., 2022). Colonisation typically begins within two years of birth and follows a dynamic and diverse course of rapid genotype turnover and simultaneous carriage of multiple strains (Faden et al., 1995; Van Kempen et al., 2001; Mukundan et al., 2007; Puig et al., 2014). NTHi is a common cause of opportunistic mucosal infections including sinusitis, paediatric conjunctivitis and community-acquired pneumonia (Van Eldere et al., 2014; Cerquetti and Giufrè, 2016). The post-Hib vaccine era has also seen a steady global increase in the incidence of invasive infections among young children and the elderly (from 10-17% in 1989 to 84-90% in 2009-2015) caused by NTHi (Ladhani et al., 2010; Cheong et al., 2015; Collins et al., 2015; Whittaker et al., 2017; Giufrè et al., 2018; Soeters et al., 2018). The highest NTHi morbidity is seen in two clinical settings: otitis media (middle ear infection) in children, and individuals with chronic lung disease (Van Eldere et al., 2014; Cerquetti and Giufrè, 2016).

### 1.1 Otitis media

OM is an important disease in early childhood globally - it is one of the most common reasons for health care visits and antibiotic prescription, and the leading cause of conductive hearing loss in children (Monasta et al., 2012). OM incidence varies geographically with recent global epidemiological estimates reporting ~391–709 million cases of OM each year (Monasta et al., 2012; Huang et al., 2025), with approximately 83% of children experiencing at least one episode by the age of three (Teele et al., 1989; Sierra et al., 2011). When taking into account recurrent episodes and seasonal variation, NTHi accounts for approximately 60% of cases (Haggard, 2008; Barkai et al., 2009). OM occurs when bacteria within the nasopharynx ascend the eustachian tube and gain access to the middle ear space (Kaur et al., 2011). Compared to other otopathogens, NTHi-associated OM is associated with higher disease severity, treatment failure, recurrence, persistence and need for repeat surgery (Barkai et al., 2009; Seppanen et al., 2020). Recurrent episodes occur in 26-54% of cases (Teele et al., 1989; Leibovitz, 2008; Arguedas et al., 2010) and greatly increase a child’s risk of developing chronic suppurative otitis media (CSOM); a complication characterised by chronic inflammation of the middle ear cavity with recurrent discharge through tympanic perforation (Welp and Bomberger, 2020). Hearing loss and consequential impairment of childhood psychosocial and cognitive development are common complications of CSOM in children (Monasta et al., 2012) and although rare, neurological sequelae account for 21,000 deaths annually (Monasta et al., 2012; Penido et al., 2016).

### 1.2 Chronic lung disease

NTHi is the leading bacterial cause of infectious exacerbations in adults with chronic obstructive pulmonary disease (COPD) and the second leading cause of infections in paediatric cystic fibrosis (Finney et al., 2014; Marshall et al., 2022). Due to a combination of host and microbial factors, NTHi persists in the lower airways of these individuals which is associated with an increased frequency of exacerbations, worsening symptoms, inflammation causing tissue damage, compositional changes to the lung microbiome and overall worse clinical prognosis (Sethi and Murphy, 2001; Sriram et al., 2017; Welp and Bomberger, 2020; Dicker et al., 2021). Similarly, children with protracted bacterial bronchitis (a disease caused by chronic infection of the conducting airways) who are positive for NTHi have reduced lung function (Craven and Everard, 2013) and are 7-fold more likely to progress to bronchiectasis within 2 years (Wurzel et al., 2016). Children with bronchiectasis experience significant morbidity and, if untreated, have poorer clinical outcomes in later life compared to patients with adult-onset bronchiectasis (King et al., 2009). As such, NTHi is an important contributor to the functional decline, disease progression, morbidity and mortality in paediatric, and adult chronic airway diseases (Sethi and Murphy, 2001).

## 2 Current management of NTHi infections necessitates alternative therapeutic strategies

### 2.1 Antibiotic therapy

NTHi-associated lower respiratory infections are typically treated with moderate spectrum β-lactam (typically amoxicillin or amoxicillin-clavulanic acid) antibiotics (Marchant et al., 2024). In COPD patients where an infection is suspected, empiric amoxicillin and doxycycline (in addition to oral corticosteroids depending on disease severity) are recommended (Yang IA et al., 2024); however, antibiotic selection may be based on local susceptibility patterns (Wedzicha et al., 2017). Although macrolides (e.g. azithromycin) have good efficacy against common respiratory pathogens and desirable anti-inflammatory properties, their use is cautioned due to potential significant adverse effects (Ni et al., 2015; Maddi et al., 2017; Yang IA et al., 2024). Amoxicillin treatment is strongly recommended for persistent OM (with or without effusion) among high-risk children (Leach et al., 2021), with amoxicillin-clavulanic acid or 2^nd^-3^rd^ generation cephalosporins (e.g. cefuroxime, ceftriaxone, cefpodoxime) being recommended where initial treatment fails (Siddiq and Grainger, 2015). Best practice guidelines for Australian First Nations children recommend CSOM is treated with ear cleaning plus topical ciprofloxacin 2–3 times per day until the ear has no discharge for 3 consecutive days (Leach, 2020). Azithromycin is recommended for acute OM cases where adherence is difficult or there is no access to refrigeration (Leach et al., 2021), and cefdinir or co-trimoxazole have been recommended for children allergic to penicillin (Siddiq and Grainger, 2015; Leach et al., 2021).

There is a growing frequency of antibiotic treatment failure among patients with NTHi-associated infections, largely owing to a rapidly evolving resistance profile (Sriram et al., 2017). In 2017, NTHi resistance was highlighted in the World Health Organisation’s list of “priority pathogens” for which new therapeutic/preventative agents are urgently needed (Organization WH, 2017). However, there have since been no advances in NTHi-targeted therapeutics and the bacterium remains on this list as of 2024 (Organization WH, 2024). The prevalence of amoxicillin/ampicillin resistance varies markedly between regions and has grown substantially over the past decade from 20-44% between 1997-2010 (Ito et al., 2010; Dabernat and Delmas, 2012; Resman et al., 2012) to 19-74% in 2016-2021 (Wang et al., 2019; Li et al., 2020; Zhou et al., 2022; El Nouwar et al., 2025). More recently, studies have reported the emergence of treatment failure with amoxicillin-clavulanic acid and cephalosporins used to treat NTHi-associated OM and community-acquired pneumonia (Patel et al., 1995; Dagan et al., 1997; Puig et al., 2015). Although global isolation of these strains is currently low (12-17%) (Dabernat and Delmas, 2012; Heliodoro et al., 2020; Li et al., 2020), their prevalence is increasing, particularly in regions such as Japan and Taiwan (Skaare et al., 2014; Honda et al., 2018; Yamada et al., 2020; Abavisani et al., 2024). Additionally, mechanisms of acquired resistance to macrolides and fluoroquinolones have also been reported, and multidrug-resistant NTHi isolates have been recovered from blood, middle ear, sputum, and nasopharyngeal specimens (Pérez-Vázquez et al., 2004; Phaff et al., 2006; Pfeifer et al., 2013; Ni et al., 2015; Puig et al., 2015; Su et al., 2020; Abavisani et al., 2024). Although currently uncommon, these resistance mechanisms have the potential to spread owing to the ability of NTHi to transfer resistant genes on mobile genetic elements and by chromosomal recombination (Hegstad et al., 2020).

The growing prevalence and spectrum of NTHi antibiotic resistance necessitates changes to antibiotic treatment guidelines that carefully balance intended clinical outcomes with the risk of further promoting antibiotic resistance. Extremely high antibiotic prescription rates are associated with the management of OM and exacerbations of COPD, a high proportion of which are considered unnecessary (Turnidge and Meleady, 2017). Antibiotic therapy may reduce middle ear damage in some patients with acute OM (Venekamp et al) but in 60% of cases, the infection spontaneously clears without antibiotic intervention and without complications (Venekamp et al]; Haggard, 2008). Similarly, antibiotic management reduces exacerbation frequency in COPD patients (Roede et al., 2009; Rothberg et al., 2010) but has no overall impact on airway destruction and disease progression (Dixit et al., 2016). In addition, NTHi frequently evades antibiotic clearance through the formation of biofilm communities or invasion of host cells (García-Cobos et al., 2014; Duell et al., 2016). For these reasons, antibiotic treatment is often insufficient to prevent relapse caused by persistent bacterial communities and does not offer long-term protection against reinfection with different strains, particularly in COPD airways (Leibovitz, 2008; García-Cobos et al., 2014; Miravitlles and Anzueto, 2015; Sriram et al., 2017).

### 2.2 Preventative strategies

In addition to the aforementioned treatment challenges, single courses of antibiotics cannot prevent reinfection and there is currently no effective NTHi vaccine. Despite the enormous success of the Hib-conjugate vaccine in preventing invasive infections caused by capsular type b *H.influenzae*, it has no effect on NTHi because these strains lacks the type b capsule (the vaccine target) (Hariadi et al., 2015). Although other vaccines comprising different antigen targets have been developed, none have proven effective in preventing infections caused by NTHi (Smith-Vaughan et al., 2014). Challenges in developing an effective vaccine arise from the enormous genetic heterogeneity among NTHi strains and the high rates of phase-variable expression of many putative vaccine targets (Murphy, 2015; Jalalvand and Riesbeck, 2018; Novotny et al., 2019). The only vaccine that has shown any potential protection against NTHi disease is the ten-valent pneumococcal conjugate vaccine containing protein D (NTHi antigen) as a carrier protein (PHiD-CV; Synflorix™, GSK Vaccines) licensed in 2008 by a number of countries for active immunisation against acute OM caused by NTHi (Clarke et al., 2017). However, randomised controlled trials of PHiD-CV among infants in Finland and Australian First Nations populations found no significant impact on NTHi carriage rates or development of OM (Vesikari et al., 2016; Beissbarth et al., 2021).

## 3 Host-NTHi competition for haem-iron

Iron is an essential micronutrient for pathogen and host alike, with critical roles in many vital cellular processes, both as an inorganic ion and through incorporation into haem, which is a molecule composed of a protoporphyrin IX (PPIX) ring and a single central iron atom (Parrow et al., 2013; Barber and Elde, 2015). Biochemical (White and Granick, 1963) and genomic studies (Cavallaro et al., 2008) indicate that NTHi lack multiple enzymes required for biosynthesis of PPIX. NTHi possesses a ferrochelatase (encoded by *hemH*), allowing it to catalyse the insertion of iron into PPIX (Schlör et al., 2000); however, there is no significant source of free PPIX in host tissues and *hemH* is not required for host colonisation or infection (Cavallaro et al., 2008), hence it appears that the growth requirement for haem must be fulfilled by acquisition of intact haem from the host. The redox potential of iron underpins both its catalytic utility in biological reactions, and production of cell-damaging free radicals. The host must therefore maintain tight regulation of systemic and cellular iron homeostasis to simultaneously meet the body’s iron demand, prevent cellular toxicity, and withhold nutrients from invading pathogens (Cassat and Skaar, 2013).

### 3.1 Haem-iron regulation in the respiratory tract

**Figure 1:**
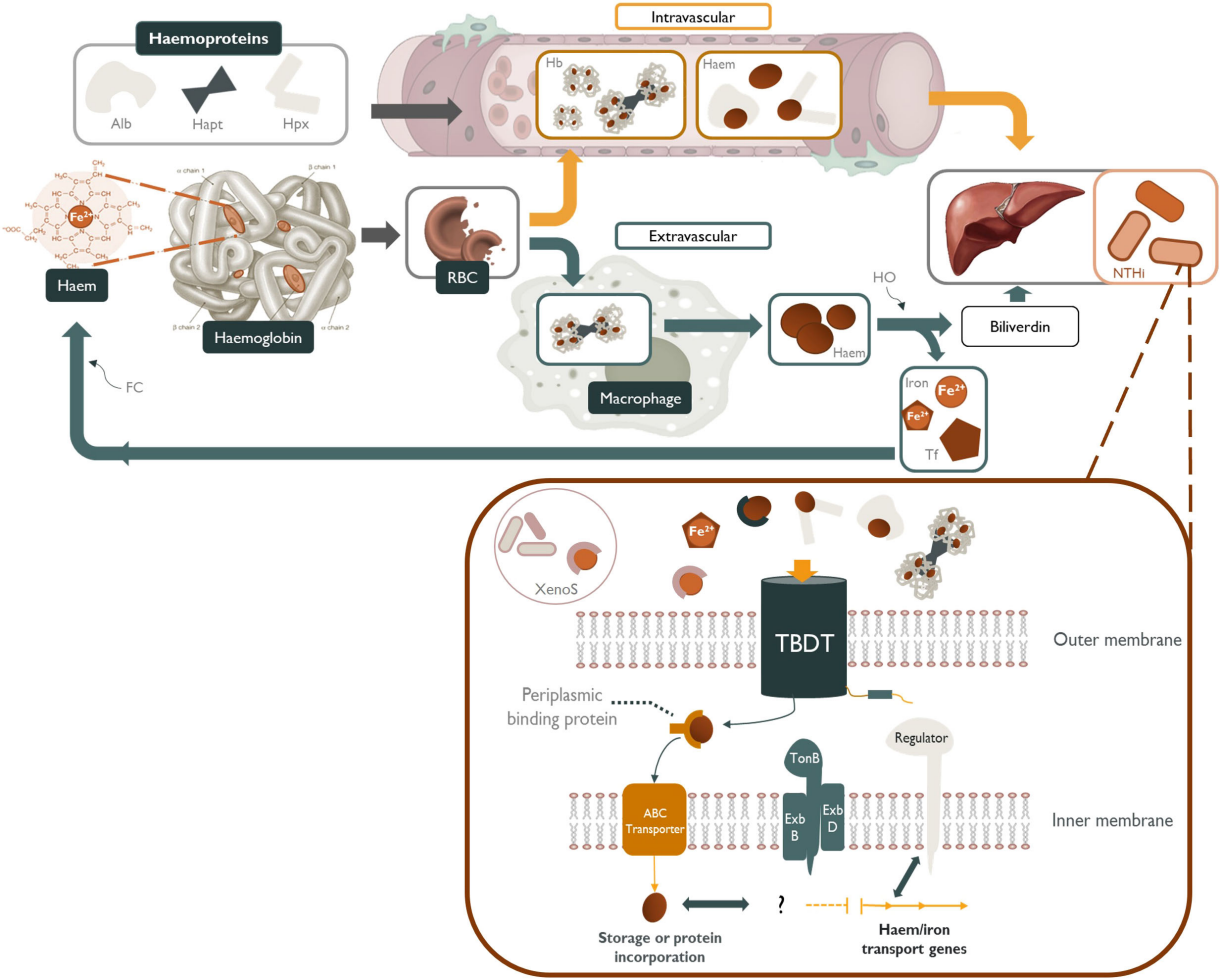
NTHi sequestration of host-derived sources of haem and other iron-containing moieties. Haem-iron released into the plasma by erythrocyte (RBC) senescence is quickly scavenged by haemoproteins such as, albumin (Alb) and hemopexin (Hpx). Any haemoglobin (Hb) released into the serum is tightly bound by haptoglobin (Hapt) and subsequently cleared by tissue macrophages (Szelestey et al., 2013; Hariadi et al., 2015). Free-haem, free-iron, transferrin-bound (Tf) iron, xenosiderophore-bound (XenoS) and haem-containing haemoproteins are readily available to NTHi as sources of nutritional haem/iron. These iron-containing molecules are transported across the periplasmic space by specific TonB-dependent transporters (TBDT) powered by a cytoplasmic transmembrane protein complex (TonB, ExbB and ExbD), followed by transport across the inner membrane by an ATP-binding cassette transporter (ABC transporter). Haem/iron acquisition in NTHi is regulated in accordance with environmental cues by iron-repressive regulators such as *fur*.

Sequestration of free iron is achieved by iron-binding proteins (such as ferritin, transferrin and lactoferrin) and by capturing haem within haemoproteins (Cassat and Skaar, 2013; Barber and Elde, 2015) (**Figure 1**). The majority of total iron in the body is found complexed as haemoglobin within erythrocytes (~1.5–2 g iron), whereas the major store of inorganic iron is ferritin, found within hepatocytes (~1 g) and macrophages (~600 mg) (Szelestey et al., 2013; Hariadi et al., 2015). Plasma iron concentrations are maintained at a low level (as complexes with transferrin), and this decreases substantially during the acute phase response to infection or other inflammatory stimuli (Ganz, 2018; Saini et al., 2024). The intracellular sequestration and the paucity of free extracellular haem-iron ensures that there is scarce availability of this essential nutrient for pathogens (Cassat and Skaar, 2013).

Iron levels are suspected to be low in the upper respiratory tract, with an estimated daily exposure of approximately 10-25 μg or around 1/1000th of that encountered by the gastrointestinal tract (Ali et al., 2017; Cloonan et al., 2017). Iron may be sourced from the pulmonary vasculature in free-, or transferrin/lactoferrin-bound forms, or as a component of haemoprotein-bound haemoglobin/haem. The high abundance of transferrin present in bronchoalveolar lavage fluid indicates that iron regulation in the lung may be primarily controlled by transferrin (Ali et al., 2017). Both alveolar macrophages and the bronchial/alveolar epithelia are able to sequester iron by various mechanisms including receptor-mediated uptake of ferric haem-iron (followed by storage within ferritin) and endocytosis of haemoglobin-haptoglobin complexes (Smith and McCulloh, 2015; Cloonan et al., 2017). The upper respiratory tract may also be exposed to inhaled atmospheric iron and iron-containing particulate matter; however, little is known about the bioavailability of this source to the host or upper respiratory microbiota (Cloonan et al., 2017).

### 3.2 NTHi acquisition of host-derived haem-iron sources

Despite the inherently low availability of haem-iron in the respiratory tract, NTHi is adept at scavenging a variety of host-derived haem-iron sources from intracellular and extracellular reservoirs, using a highly complex and redundant repertoire of haem acquisition systems (**Table 1**). These mechanisms capture iron/haem molecules that are free in solution or complexed with host proteins and shuttle the iron/haem moiety across the cell wall and both bacterial membranes in a series of protein-ligand interactions (**Figure 1**) (Cassat and Skaar, 2013). NTHi is also capable of storing excess haem which creates an intracellular surplus that can be donated to starved bacterial cells in haem-iron deplete conditions; however, the mechanisms are currently unknown (Rodríguez-Arce et al., 2019).

**Table 1:** NTHi proteins and regulatory elements involved in the acquisition of host-derived haem-iron and non-haem iron sources *in vivo*, and evidence for their potential roles in pathogenesis.

System/Protein (Function, cell location)	Haem/Iron Sources	Proposed Role in Pathogenesis
Haem acquisition systems		
HxuCBA System - HxuA (haemophore, OMP) - HxuB (transporter, OMP) - HxuC (receptor, PP)	Haem: free, hpx-, alb- Hb: free, hapt-	Colonisation: Mediates adherence to cultured airway epithelia (Seale et al., 2006; Morton et al., 2007) Virulence: Higher prevalence in middle ear strains (Hariadi et al., 2015); establishment of invasive disease and mortality in rats (Seale et al., 2006; Morton et al., 2007) Persistence: Potentiates antibiotic resistance (Rodríguez-Arce et al., 2019)
HgpBCD - HgpB (receptor, OMP) - HgpC (receptor, OMP) - HgpD (receptor, OMP)	Hb: free, hapt-, mb-	Virulence: Higher prevalence in middle ear strains (Hariadi et al., 2015); establishment of invasive disease and mortality in rats (Seale et al., 2006; Morton et al., 2007) Survival: Required for bacterial proliferation during NTHi-induced OM in chinchillas (Morton et al., 2004)
HemR (receptor, OMP)	Haem: free	Virulence: Polymorphisms associated with OM isolates, compared to commensal isolates (LaCross et al., 2014)
HhuA (receptor, OMP)	Hb: hapt-	Survival: Required for growth *in vitro* (Maciver et al., 1996)
TehB (unknown)	Haem: free, alb- Hb: free, hapt-	Virulence: required for establishment and persistence of bacteraemia in rat models of *H. influenzae* invasive disease (Whitby et al., 2010)
Iron acquisition systems		
TbpAB - TbpA (iron sequestration, OMP) - TbpB (co-receptor, OMP)	Iron: transferrin	Virulence: Conserved in all invasive NTHi isolates (Gray-Owen and Schryvers, 1995); upregulated during lung infection (Polland et al., 2023)
FbpABC (HitABC) - FbpA (ion-binding, PP) - FbpB (transporter, IMP) - FbpC (transporter, IMP)	Iron: transferrin	Colonisation: Adhesion molecule of *Listeria monocytogenes* (Osanai et al., 2013); no data available for NTHi Virulence: upregulated during lung infection (Polland et al., 2023)
Fhu - FhuB (binding protein, PP) - FhuCD (permease, IMP)	Xenosiderophore acquisition	Survival: May utilise siderophores made by other bacteria (Morton et al., 2010a)
Haem-iron transport/storage proteins		
Protein E (storage/donation, OMP)	Haem: free	Colonisation: Adherence to cultured and mouse lung epithelia (Singh et al., 2010; Duell et al., 2016) Survival/persistence: Invasion of cultured and mouse bronchial epithelia (Ikeda et al., 2015); potentiates antibiotic resistance (Rodríguez-Arce et al., 2019); inter-bacterial donation of haem (Singh et al., 2010; Duell et al., 2016)
SapABCDFZ (transport, PP)	Haem: free	Colonisation/persistence: Adhesion, colonisation and biofilm formation in chinchilla middle ear (Raffel et al., 2013; Clementi et al., 2014) Virulence: Establishment of chinchilla OM (Raffel et al., 2013; Clementi et al., 2014)
HbpA-DppBCDF (transport, PP)	Shuttles haem from any source	Virulence: Establishing bacteraemia in a mouse model (Rosadini et al., 2008) Persistence: Mediates glutathione import and antibiotic resistance (Vergauwen et al., 2010; Rodríguez-Arce et al., 2019) Survival: Important for periplasmic transport of haem through other outer membrane channels (Tanaka and Pinkett, 2019)
Hup (accessory/transport for Hgp and Hxu systems, OMP)	Haem: free, hpx-, alb- Hb: free, hapt-	No data available about role in pathogenesis
Haem-iron Regulatory Elements		
*fur*	Master regulator of genes/proteins involved in haem-iron utilisation	Virulence/persistence: Critical for bacterial virulence and persistence in a mammalian model of OM (Harrison et al., 2013); upregulates IgA1 protease which mediates antibiotic resistance, optimal invasion and long-term intracellular survival in human respiratory epithelial cells (Clementi et al., 2014) Colonisation: Additional regulatory management of molecules shown to mediate attachment (Harrison et al., 2013)
*icc*	Stress response to transient haem/iron limitation	Virulence/persistence: Mutations result in epigenetic and morphological adaptations that contribute to persistence and disease severity in experimental models of OM (Szelestey et al., 2013; Hardison et al., 2018b)
*modA*	Downstream phase-variable regulation of haem/iron acquisition proteins	Virulence/persistence: Phase variation of antibiotic resistance, biofilm formation and immunoevasion; biphasic switching during chinchilla OM (Atack et al., 2015a)

Hb, Haemoglobin; hapt-, haptoglobin-bound; alb-, albumin-bound; haemopexin-bound, hpx-; mb-, myoglobin-bound; hxu, haem/hemopexin utilisation protein; Tbp, transferrin-binding proteins; Hgb, haemoglobin binding protein; HemR, haem receptor; Hup, haem-utilisation protein; Hhu, haemoglobin-haptoglobin binding protein; FbpA, ferric-binding protein; Fhu, ferric hydroxamate uptake; Sap, sensitivity to antimicrobial peptide; IgA1, immunoglobulin A1; HbpA, haem-binding lipoprotein; *fur*, ferric uptake regulator; TehB, tellurite-resistance determinant; OMP, outer membrane protein; PP, periplasmic protein; IMP, inner membrane protein.

Haemoprotein-mediated sequestration (as opposed to acquisition of free iron) from the extracellular environment is the most common mode of iron acquisition used by NTHi. Target-specific surface-anchored proteins function by taking up free haem or extracting it from haemoproteins in the extracellular medium and delivering it to a TonB-dependent outer membrane receptor (Hariadi et al., 2015). Quantitatively, haemoglobin and haemoglobin-haptoglobin complexes are likely to be the most significant extracellular sources of haem *in vivo* (Seale et al., 2006). This is reflected by the sheer number of mechanisms possessed by NTHi for uptake of these molecules and the attenuated pathogenesis of strains unable to utilise them (Morton et al., 2004). Although less common, NTHi (approximately 3% of isolates) also express receptors that directly recognise and extract iron from transferrin and/or lactoferrin (Barber and Elde, 2015). In other bacterial species, the most common strategy for obtaining iron from these molecules involves the secretion of siderophores; small-molecule ferric chelators which sequester iron from host transferrin (Bullen et al., 1999). Unlike most bacteria, NTHi does not possess genes encoding proteins for siderophore synthesis; however, an iron-repressible siderophore utilisation locus was discovered in several NTHi strains that may enable utilisation of xenosiderophores produced by other microorganisms *in vivo* (Morton et al., 2010a). Such a tactic would further expand the pool of iron sources available to NTHi, without the energy burden associated with siderophore synthesis (Hariadi et al., 2015).

Once complexed by haem-targeting proteins or xenosiderophores, a set of TonB-dependent transporters (TBDTs) are required to transport the haem/iron into the periplasmic space, followed by transport across the inner membrane by ATP-binding cassette (ABC) transporters (Sgheiza et al., 2017). A cytoplasmic transmembrane protein complex composed of three proteins, TonB, ExbB and ExbD, spans the periplasm and interacts with specific TBDTs. This TonB complex transduces the proton motive force of the cytoplasmic membrane to energise transport of substrates through a specific TBDT (Noinaj et al., 2010). Once inside the cell, the expectation is that haem-bound iron can be liberated through enzymatic cleavage of the porphyrin ring by haem oxygenase enzymes that are present in many bacterial species (Richard et al., 2019). Haem-iron acquisition in NTHi is regulated in accordance with environmental iron-bioavailability by iron-repressive transcriptional regulators such as *fur*, with the result that iron uptake is coordinated with storage and efflux to ensure iron homeostasis (Cassat and Skaar, 2013). Tight regulation of haem-iron uptake is critical for balancing the metabolic requirement with the potentially toxic consequences of excess iron (Harrison et al., 2013).

### 3.3 Dysregulation of host haem-iron homeostasis

High iron availability has been shown to increase the pathogenic potential of many bacteria in tissue and animal infection models by enhancing growth, cellular adhesion, invasion and epithelial damage (Reid et al., 2009; Kortman et al., 2012; Nairz et al., 2017). Thus, disorders that interfere with iron-restricting host responses may predispose individuals to NTHi infections. High susceptibility to infection with a variety of bacterial genera, including *Haemophilus*, has been described in several iron overload disorders, such as hereditary haemochromatosis, β-thalassemia, sideroblastic anaemia, transfusion-dependence, or chronic liver disease (Weinberg, 2000; Rahav et al., 2006; Kontoghiorghes et al., 2010; Quenee et al., 2012). In these conditions, the iron-binding capacity of serum transferrin is exceeded, resulting in low affinity complexes of iron with other plasma components (Cassat and Skaar, 2013), thus providing an easily accessible iron source for microbes. Iron overload is also associated with defective chemotaxis and phagocytosis by neutrophils and macrophages, as well as decreased bactericidal activity that contributes to decreased immune function (Porto and De Sousa, 2007; Cherayil, 2010). Thus, a combination of impaired immune function and increased iron availability may contribute to the heightened susceptibility to infection in conditions of iron-overload.

High levels of iron and/or ferritin have been detected in the airways of individuals with COPD and cystic fibrosis, and in heavy smokers, suggesting dysregulation of iron homeostasis in these conditions (Stites et al., 1999; Ali et al., 2017). In these conditions, excess iron appears to reside intracellularly in alveolar macrophages and, to a lesser extent, within the epithelial lining fluid of the lung (Mateos et al., 1998). While these findings suggest a role of excess iron in the characteristically high predisposition to NTHi infections observed in these patient groups (Fenker et al., 2018), correlative studies are required to determine if this is the case. Similarly, geogenic particles from remote regions of Australia (communities with a disproportionate burden of respiratory disease and infection) with high iron oxide levels were shown to increase NTHi invasion of bronchial epithelial cells *in vitro* compared to low iron oxide particulate matter from other regions (Williams et al., 2023). However, further studies are required to determine whether there is a direct correlation between NTHi disease prevalence and local geogenic iron oxide particulate levels among the broader population.

## 4 Haem-iron is an important mediator of NTHi pathogenesis and persistence

**Figure 2:**
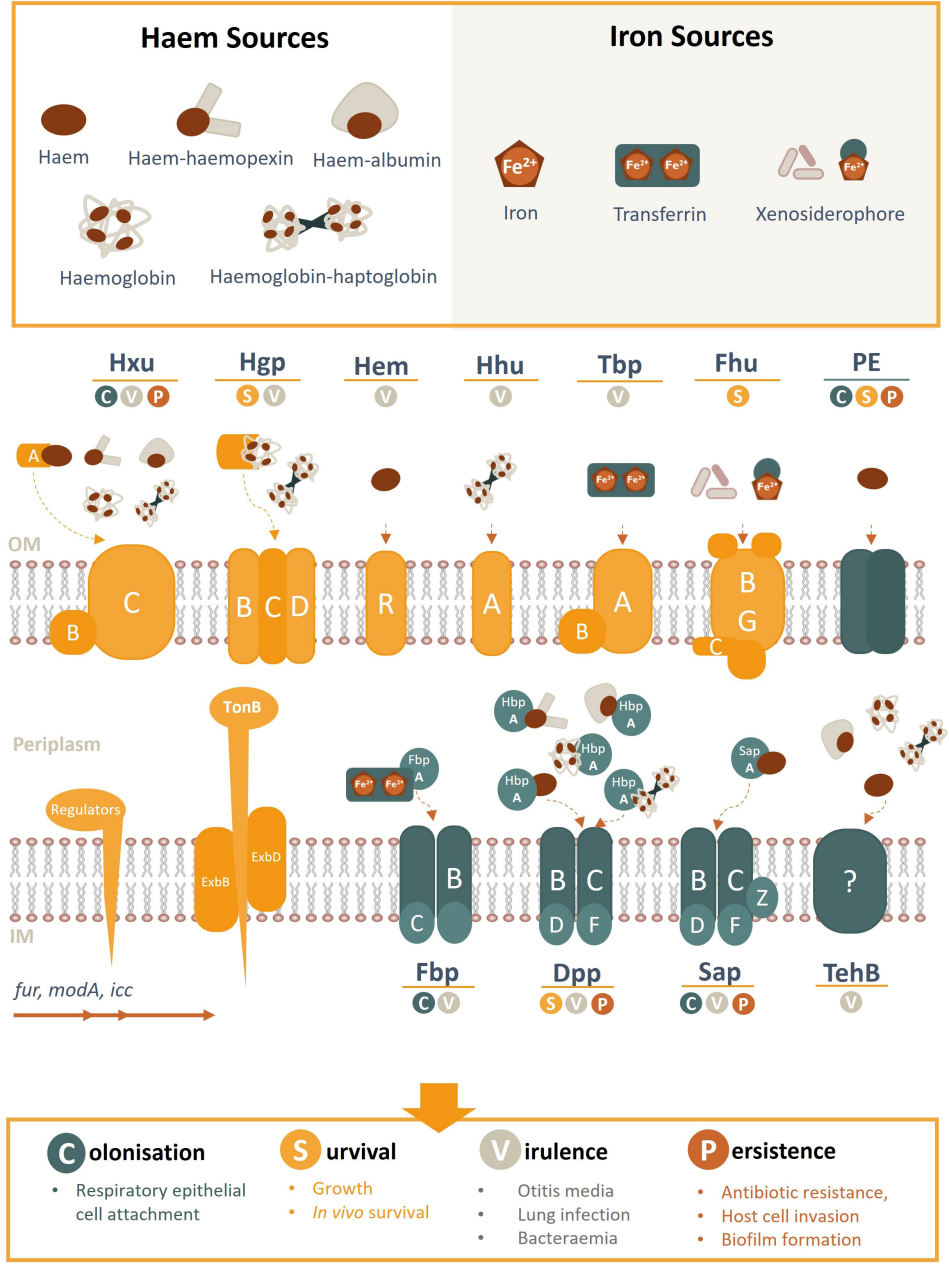
Schematic overview of NTHi haem/iron uptake systems and transporters, their cognate haem/iron sources, and role in pathogenesis. Known haem acquisition systems include HxuABC, HgpBCD, HemR and HhuA. The Hxu system involves the HxuAB two-partner secretion system whereby HxuA is exposed at the cell surface and leads to haem release and its capture by the HxuC receptor. The Hgp system involves haem sequestration by a secreted haemophore which is transported across the outer membrane by the HgpBCD receptor. Outer membrane receptors HemR and HhuA bind directly to host haem or haemoproteins. Iron uptake (free- or transferrin-bound) is coordinated by TbpAB, Fhu (xenosiderophores) and FbpABC (HitABC). Once within the periplasm, haem/iron are transported across the inner membrane by ABC transporters (e.g. Fbp, DppBCDF, SapBCDFZ and TehB) by their periplasmic proteins (e.g. SapA, HbpA, FbpA). PE is an outer membrane protein that binds haem as a dimer. Regulatory gene elements are indicated and systems highlighted in yellow are TonB-dependant (opposed to blue TonB independent receptors). OM, outer membrane; IM, inner membrane.

The success of NTHi as a pathogen is reliant on its ability to perform interactions with the host, many of which are regulated directly or indirectly by the bioavailability of haem or other iron-containing moieties (Harrison et al., 2013). Haem-iron acquisition is coordinated by the ferric uptake regulator *fur*; a master regulator of genes involved in the uptake of iron and iron-containing moieties in many bacterial species (Hassan and Troxell, 2013). Additional regulatory management of NTHi-host cell interactions by *fur* have been reported (Hassan and Troxell, 2013). Haem and iron acquisition systems have also demonstrated a role in sensing host environmental cues to mediate interactions with host epithelial cells. As such, the regulatory feedback between haem/iron sequestration and interactions with the host plays an important role in all facets if NTHi pathogenesis (Harrison et al., 2013; Rodríguez-Arce et al., 2019). Haem/iron acquisition systems and evidence for their role in NTHi pathogenesis are summarised in **Table 1**, **Figure 2**.

### 4.1 Host attachment and colonisation

In all disease contexts, nasopharyngeal colonisation by NTHi is a critical antecedent of subsequent ear and lower airway infections (Hare et al., 2010; Duell et al., 2016). Additionally, a higher strain turnover and density of NTHi colonisation has been linked with an in increased risk of developing OM (Harabuchi et al., 1994; Smith-Vaughan et al., 2006; Kirkham et al., 2010; Smith-Vaughan et al., 2013), acute exacerbations in COPD (Van Kempen et al., 2001; Sethi et al., 2002) and lower airway infections in children with bronchiectasis (Hare et al., 2010). For this reason, populations with high NTHi carriage rates experience the highest OM disease burden (Sun et al., 2012; Coleman et al., 2018). Likewise, among patients with COPD, nasopharyngeal acquisition of a new NTHi strain is frequently associated with the onset of an acute exacerbation (Sethi et al., 2002). Furthermore, elevated nasopharyngeal NTHi bacterial load can result in a clinically significant increase in COPD symptoms, even in the absence of a clinical exacerbation (Pettigrew et al., 2012).

Successful host colonisation is achieved by a suite of outer membrane proteins that mediate attachment to the nasopharyngeal epithelium (Atto et al., 2019). Upregulation of proteins involved in host-cell adhesion has been demonstrated in iron depleted media designed to replicate respiratory tract conditions (Pidcock et al., 1988; Qu et al., 2010), suggesting that iron bioavailability influences adhesin expression. Transcriptomic and proteomic analysis of *fur* has revealed additional regulatory management of several molecules that can mediate attachment and colonisation, such as the high-molecular weight proteins (HMW) (Qu et al., 2010; Harrison et al., 2013). HMW1 and HMW2 are major NTHi adhesins present in 75-80% of isolates (Buscher et al., 2004). These glycopeptides bind to integrin-receptors on epithelial cell surfaces and are vital to the bacterium’s ability to adhere to cultured human respiratory epithelial cells and to colonise the upper respiratory tract of rhesus macaques *in vivo* (Rempe et al., 2016).

Interestingly, some proteins involved in host-cell colonisation have demonstrated moonlighting functions directly involved in haem or iron acquisition. The outer membrane adhesin, protein E (PE), is ubiquitous among NTHi clinical isolates and hijacks host vitronectin-integrin binding to promote cell adhesion (Ikeda et al., 2015). The requirement for PE in the optimal adherence and persistence of NTHi within the airways has been demonstrated in mice immunised with anti-PE antibodies (Ronander et al., 2009). More recently, PE was shown to form high affinity interactions with haem and influence the ability of *H. influenzae* to acquire haem *in vitro*. PE-bound haem was also donated to haem-starved populations during co-culture, suggesting a secondary role for PE as a haem storage site that could be later distributed to nearby starved cells and promote survivability under conditions of fluctuating haem availability (Al Jubair et al., 2014; Rodríguez-Arce et al., 2019). The Sap (sensitivity to antimicrobial peptide) inner membrane ATP-binding-cassette (ABC) transport complex also appears to be important in the ability of NTHi to acquire haem-iron and colonise the host (Mason et al., 2011). Deletion of the Sap structural ATPase protein, SapF, simultaneously inhibits recovery of depleted internal haem-iron stores and reduces the ability of NTHi to colonise the nasopharynx or cause acute infection in a chinchilla model of acute OM (Vogel et al., 2012). The periplasmic binding protein, SapA was also found to be essential for haem transport and utilisation by haem-starved NTHi (Mason et al., 2011) and adherence to polarised epithelial cells through host environmental cues (Raffel et al., 2013). The shared host-cell adhesion and haem-binding functionality of many NTHi proteins suggests that bacterial haem-utilisation plays an important role in its interplay with host epithelial cells and may provide an adaptive advantage for colonisation in environments with low haem-iron availability (Ghio, 2009).

### 4.2 Survivability and virulence in the respiratory tract

Continued NTHi survival in the respiratory tract requires a secure source of host-derived haem; accordingly, strains with a reduced capacity to acquire haem were shown to have a substantially shortened lifespan in broth culture and in chinchilla airways (Maciver et al., 1996; Morton et al., 2004). The higher prevalence of multiple haem-acquisition genes in disease-associated isolates compared to throat colonising strains or the non-pathogenic relative *H. haemolyticus* is consistent with the notion that the ability to acquire diverse haem sources provides an adaptive advantage in pathogenic isolates (Qu et al., 2010; Hariadi et al., 2015). A conserved genetic island unique to disease-associated NTHi features an overrepresentation of genes associated with haem acquisition and transport (Zhang et al., 2012). Inactivation of multiple genes associated with haem-utilisation was found to attenuate NTHi virulence-determinants and disease severity/duration in animal models of acute OM and lung infection (Morton et al., 2004; Morton et al., 2009; Szelestey et al., 2013; Rodríguez-Arce et al., 2019). Similarly, an isogenic mutant of two haem-acquisition pathways was unable to sustain bacteraemia or produce meningitis in a rat model of invasive disease (Seale et al., 2006). Collectively, these data indicate that iron-dependent *fur-*regulated genes contribute to longer and more severe infections by regulating iron within the cell (Ahearn et al., 2017).

The ability to acquire specific haem sources may also be linked to the pathogenic potential of NTHi isolates. Capacity to utilise iron from transferrin or from haptoglobin-bound haemoglobin has been associated with invasive *Haemophilus* spp. isolates (Hardie et al., 1993; Zhang et al., 2012) and is not an ability shared among non-pathogenic members of the *Haemophilus* genus (Williams et al., 1990). Additionally, NTHi strains deficient in the ability to acquire haemoglobin-haptoglobin have an attenuated ability to cause infection (Seale et al., 2006). Individual NTHi strains are capable of producing up to four different haemoglobin/haemoglobin-haptoglobin binding proteins (Hgp) that collectively display affinity for all known human haptoglobin phenotypes (Morton et al., 2006). The presence of multiple *hgp* genes may allow for selective expression of the Hgp with greatest affinity for the predominant host haemoglobin-haptoglobin phenotype, or alternatively as a strategy to evade the host immune response mounted against a particular Hgp-expressing population (Langlois and Delanghe, 1996). There are 2 major haptoglobin alleles (hp1 and hp2), with each allele coding for a subunit capable of binding one haemoglobin αβ dimer. The Hp1/2 proteins form different oligomers, such that a dimeric Hp1–1 molecule can bind two haemoglobin αβ dimers, while the polymeric Hp2–1 and Hp 2–2 molecules have the capacity to bind greater numbers of haemoglobin αβ dimers (Morton et al., 2006). The prevalence of expressed haptoglobin phenotypes varies between populations, with the Hp2–2 phenotype predominating in Indian and Australian First Nations populations (Langlois and Delanghe, 1996). A growth preference for the Hp 1–1 phenotype has been demonstrated by *H. influenzae in vitro* (Morton et al., 2006); however, the correlation with susceptibility to infection *in vivo* has not been investigated. This also highlights the need for consideration of haem source in *in vitro* models, where free haem is typically added as the sole source in culture media: a condition which may not reflect predominant sources in the respiratory and middle ear niches. For example, the iron-containing moiety lactotransferrin was more than 10-fold more abundant in middle ear effusions collected from children with chronic OM than haemoglobin (Val et al., 2016).

There is also evidence to suggest that haem-iron accessibility may influence adaptive transitions between commensal and pathogenic states. For many opportunistic pathogens, iron starvation not only triggers activation of genes involved in its uptake, but also those involved in virulence determinants (Chen et al., 2011; Hassan and Troxell, 2013; Jung and Do, 2013). In *Pseudomonas aeruginosa*, positive selection for promotor mutations that increase expression of the bacterial uptake system *phu* (pyoverdine siderophore) occurs exclusively among isolates from patients with cystic fibrosis that show enhanced bacterial growth (Marvig et al., 2014). NTHi haem-iron acquisition proteins (along with proteins associated with biofilm formation, antibiotic resistance and immune evasion) are subject to changes in gene expression, which are differentially regulated during experimental OM (Atack et al., 2015a) and in isolates recovered from COPD airways (Rao et al., 1999; Ren et al., 1999; Poole et al., 2013) by the modA phase-variable regulon (phasevarion). Switching of *modA* expression from OFF to ON state within the middle ear produces a phenotypic switch in several genes during infection that increases disease severity and permits bacterial persistence by increasing cell adhesion, invasion and robust biofilm formation (Atack et al., 2015a; Brockman et al., 2016; Brockman et al., 2017; Brockman et al., 2018). Specifically, in a *ModA16* ON state, NTHi upregulates multiple iron-acquisition factors including an iron ABC transporter substrate-binding protein, and haem/haemopexin-binding proteins HxuC and HxuB (Tram et al., 2024). Independent phase-variable expression has also been observed in genes involved in haemoglobin-haptoglobin acquisition (Phillips et al., 2022) which is hypothesised to provide an adaptive response to fluctuations in haem-iron availability/source influenced by the stage or site of infection (Atack et al., 2015b). Additionally, strand slippage may provide a mechanism to avoid the host immunological response by expressing proteins that are functionally similar, but antigenically distinct (Rao et al., 1999; Ren et al., 1999).

### 4.3 Persistence in the respiratory tract

NTHi persistence within the respiratory tract not only enables the potential for disease recurrence/relapse but also induces persistent airway inflammation and promotes disease progression in COPD lungs (Murphy et al., 2004; Finney et al., 2014; Ahearn et al., 2017). To persist within the airways, NTHi must subvert or evade clearance by antibiotics or the innate host immune response. This is achieved by a variety of mechanisms that include specific neutralisation of antimicrobial agents, resistance to nutritional immunity, invasion of host epithelial cells and formation of protective biofilms (Morton et al., 2004; King, 2012; Vogel et al., 2012). Haem-iron bioavailability has been shown to influence many NTHi behaviours associated with persistence (Szelestey et al., 2013).

An important first-line defence by the host against microbial growth is to further reduce circulating iron concentrations to limit microbial survival; a process known as nutritional immunity. Siderocalins contribute to this antimicrobial defence by sequestration of microbial siderophores (Clifton et al., 2009). NTHi uses siderophore-independent mechanisms, such as haem-uptake, to acquire iron and so siderocalins are ineffective and this may provide an adaptive advantage over competing bacteria in the respiratory tract that rely on siderophore-mediated acquisition of iron for survival (Nelson et al., 2005; Holden and Bachman, 2015). The initial immune response against bacterial infection of mucosal surfaces in the respiratory tract also involves recruitment of immune cells and abundant production of antimicrobial peptides (AMPs) (Geitani et al., 2020). IgA1 is the principal immunoglobulin subclass produced by respiratory mucosal tissues and plays a major role in host defence by inhibiting microbial adherence, inactivating bacterial toxins, and promoting humoral immunity (Geitani et al., 2020). NTHi counteracts this response by producing an IgA1 protease, an extracellular endopeptidase which specifically cleaves IgA1 (Rao et al., 1999). IgA1 protease production appears to be exclusive to pathogenic *Haemophilus* species and is upregulated by *fur* in response to iron-restricted conditions (Rao et al., 1999).

Coordination between bacterial immune evasion and haem homeostasis has been observed for several NTHi proteins involved in the acquisition/utilisation of haem. HxuCBA, SapA, and PE have been shown to confer resistance to AMP LL-37 and a homologue of human β-defensin in a chinchilla model of acute OM (Mason et al., 2005; Vogel et al., 2012; Rodríguez-Arce et al., 2019). The consequence of inhibiting AMP resistance was observed in a *sapA* mutant which had an attenuated ability to survive in both the nasopharynx and the chinchilla middle ear, compared to the parent strain (Mason et al., 2005; Shelton et al., 2011). HbpA, the substrate binding protein that transports haem within the periplasm, is responsible for transport of glutathione, a thiol-containing tripeptide that confers protection against oxidative, xenobiotic, and metal iron stresses (Vergauwen et al., 2010; Rodríguez-Arce et al., 2019). NTHi cannot synthesise glutathione and absence of glutathione from the growth medium has been shown to reduce survival of *H. influenzae* Rd in culture (Vergauwen et al., 2003). Thus, NTHi may take advantage of the same import systems to both obtain haem and confer resistance to the innate mucosal immune response and oxidative stress (Mason et al., 2006; Rodríguez-Arce et al., 2019). This may contribute to a predisposition to NTHi infections in CF and COPD patients who demonstrate compromised lower airway AMP activity despite mounting an inflammatory response (Persson et al., 2017; Geitani et al., 2020).

NTHi invasion of host epithelial cells may not only provide a means of evading host immune pressures, but also provide an alternative reservoir for nutrient acquisition when bacteria are exposed to nutrient-limiting conditions (Harrison et al., 2013). Chinchilla middle ears challenged with *sapA* mutants deficient in haem-iron uptake demonstrated a tendency towards a more persistent phenotype, favouring intracellular survival and a dampened cytokine response compared to the parent strain (Raffel et al., 2013). Similarly, expression of *fur-*regulated IgA1 protease (Harrison et al., 2013) was required for optimal invasion and long-term intracellular survival in bronchial epithelial cells (Clementi et al., 2014). While these observations suggest environments restrictive of iron promote NTHi invasion and persistence, other studies have demonstrated a more nuanced relationship between haem/iron availability and NTHi phenotype. NTHi mutants lacking *hxuCBA, hbpA, hpe* or *sapA* haem-acquisition systems had an impaired ability to invade type II pneumocytes (Rodríguez-Arce et al., 2019). Similarly, mutants lacking the conserved iron-regulon *fur* also exhibited reduced persistence in middle ears of chinchillas (Harrison et al., 2013). Two studies (Szelestey et al., 2013; Hardison et al., 2018a) have attempted to understand this discrepancy through transient haem-iron restriction of NTHi. Exposure to excess haem-iron was only found to promote a persistent phenotype in experimental OM models for NTHi strains previously starved of haem-iron, compared to those continuously exposed to haem-replete conditions (Szelestey et al., 2013; Hardison et al., 2018b). This response was attributed to microevolutions through mutations in *icc* which result in epigenetic and morphological adaptations that contribute to persistence and disease severity (Hardison et al., 2018a). Recurrent episodes in a separate pre-clinical OM model also demonstrated continuous microevolution of a haemoglobin-binding gene that resulted in a highly invasive NTHi phenotype that persisted for at least one month following clinical resolution of the infection (Harrison et al., 2020).

The ability to form sedentary communities or biofilms in anatomical sites, such as the middle ear, has also been shown to contribute to the *in vivo* persistence of NTHi (García-Cobos et al., 2014). In addition to protection from immune- or antibiotic-mediated killing, biofilm formation may also act as a mucosal reservoir for NTHi following resolution of clinical disease, thus promoting bacterial persistence and re-infection within the airways (Jung and Do, 2013; Raffel et al., 2013). Biofilm-resident bacteria exhibit a reduced metabolism and an altered proteome compared to their planktonic counterparts, features that contribute to their reduced susceptibility to immune effectors and commonly used antibiotic treatments (Novotny et al., 2019). This mechanism is hypothesised to contribute to the chronic and recurrent nature of NTHi-associated infections, including bronchitis, acute exacerbations of COPD, conjunctivitis, sinusitis, and OM (Kaya et al., 2013; Gu et al., 2014; Novotny et al., 2019; Welp and Bomberger, 2020). Haem-iron restriction or loss of the utilisation protein SapF results in morphological plasticity and enhanced community development and biofilm architecture (Vogel et al., 2012; Szelestey et al., 2013). Conversely, excess haem-iron availability was shown to increase peak height and architectural complexity of NTHi biofilms following a period of haem-iron restriction (Szelestey et al., 2013).

## 5 Disruption of haem-iron assimilation: an effective therapeutic strategy against NTHi infection?

The diminishing effectiveness of current antibiotic treatments and the challenges associated with vaccine development have encouraged exploration of novel therapeutic strategies to prevent and/or treat NTHi infections. The dependence for haem-iron at all stages of NTHi pathogenesis exposes a vulnerability that provides promising targets for the development of new therapies that may disrupt iron uptake (Stites et al., 1999; Ahearn et al., 2017). In animal models, extracellular iron restriction was shown to be effective in preventing respiratory infection and dissemination of pneumonia caused by a variety of Gram-negative bacteria, including *H. influenzae* (Minandri et al., 2016; Michels et al., 2017). Similarly, disruption of haem or iron acquisition mechanisms significantly affects the ability of NTHi to cause disease in animal models (Ahearn et al., 2017). Currently, multiple host- and bacterial-targeted approaches in development aim to disrupt haem and/or iron assimilation by a range of pathogens and may have utility against NTHi (**Table 2**).

**Table 2:** Strategies that target bacterial haem-iron assimilation and their potential application for the prevention or treatment of NTHi infections.

Strategies	Example	Antimicrobial activity	Utility against NTHi?	Challenges/limitations
Pharmaceutical Approaches				
Inhibitors of siderophore biosynthesis/ function	Flucytosine: non-ribosomal peptide synthetase (NRPS) enzymes inhibitor	Suppresses *P. aeruginosa* pathogenicity in a mouse model of pulmonary infection (Imperi et al., 2013)	Siderophore-inhibiting molecules do not disrupt the ability of *H. influenzae* to acquire haem-iron *in vivo* (Nelson et al., 2005)	Efficacy: adaptive resistance through production of structurally modified siderophores has been described (Abergel et al., 2006; Raffatellu et al., 2009; Holden and Bachman, 2015)
	Baulamycins A and B: NRPS independent siderophore synthetase enzymes	*In vitro* antibacterial activity against *S. aureus* (staphyloferrin B) and *B. anthracis* (petrobactin) (Tripathi et al., 2014)		
	Small molecule inhibitors	Mitigates *P.aeruginosa* pathogenesis in a nematode host (Kirienko et al., 2019)		
Sideromycins	Cefiderocol (Fetroja): Siderophore-mimicking compound conjugated to a cephalosporin	Bactericidal activity against β-lactamase producing and multidrug-resistant Gram-negative species *in vivo* (Matsumoto et al., 2017)	Effectiveness has only been demonstrated against NTHi *in vitro* under conditions with artificially restricted haem-iron availability (Ito et al., 2018)	Safety profile: toxic side-effects in the elderly; no safety data for children Efficacy: inhibition of siderophore-mediated iron-acquisition may be overcome by acquisition of haem Acquired resistance: has been demonstrated in gram-negative bacteria (Kohira et al., 2020)
Iron Chelators	Desferrioxamine B (DFO): intravenous iron chelator	Some antibacterial and antibiotic potentiating activity observed *in vitro* (Hartzen et al., 1994)	NTHi can utilise iron sequestered within DFO as an iron source (Williams et al., 1990)	Safety profile: may increase susceptibility to infection (Rahav et al., 2006; Lal et al., 2013) Efficacy: can be used as an iron source, which promotes growth of some bacteria (Choon-Mee et al., 2007; Thompson et al., 2012)
	Deferiprone (DFP): oral iron chelator	Activity against common Gram-negative nosocomial pathogens (Thompson et al., 2012)	Activity against NTHi has not been tested.	Efficacy: may be subverted by acquisition of haem-iron
Haem-Degradation inhibitors	Gallium-protoporphyrin IX: haem analogue	*In vitro* activity against multidrug-resistant Gram-negative species, including intracellular and biofilm communities (Arivett et al., 2015; Hijazi et al., 2017; Hijazi et al., 2018; Choi et al., 2019)	NTHi is highly reliant on exogenous haem-acquisition; activity against NTHi has not been tested	Application: no *in vivo* evidence; currently limited to topical applications
	Haem-oxygenase inhibitors	*In vitro* activity against *P.aeruginosa* (Furci et al., 2007)	Not dependent on haem-source; capable of blocking the oxidation of haem at high concentrations (Furci et al., 2007)	
Immune-based Approaches				
Vaccine targets	Protein E: Outer-membrane protein	Induces an antibody response capable of blocking haem-acquisition and epithelial adhesion	Elicits potent bactericidal immune response in mice (Behrouzi et al., 2017); a PE-PilA fusion protein protected against NTHi colonisation and biofilm integrity in the mouse nasopharynx (Ysebaert et al., 2019)	Efficacy: no protection against *H. influenzae* in the lungs of COPD patients (Wilkinson et al., 2019); high mutability, phase-variability and redundancy of NTHi -acquisition systems may limit efficacy
	Multiple haem/iron epitope targets	Protects mice from infection caused by uropthogenic *E. coli* (Alteri et al., 2009; Mike et al., 2016) and against intravenous challenge with *S. aureus* (Kim et al., 2010)	Interference of multiple haem-utilisation systems attenuates NTHi virulence and disease severity/duration in animal models of OM and lung infection (Morton et al., 2004; Morton et al., 2009; Szelestey et al., 2013; Rodríguez-Arce et al., 2019)	Application: no candidates currently available for NTHi
	Na-APR-1; hookworm haemoglobinase	Significantly reduced parasite burden in experimentally-infected canines (Cassat and Skaar, 2013)	No antibacterial activity	Application: development of a similar strategy requires full elucidation of mechanisms governing NTHi haem-utilisation
Bacteriotherapy				
Bacteriocin-producing probiotics	Haemophilin-producing *H. haemolyticus*	Inhibits NTHi growth (Latham et al., 2017) and interactions with host cells (Atto et al., 2021a)	Blocks NTHi access to haem; as a commensal of the same family, Hh is likely to have highly potent anti-NTHi activity	Application: requires further investigation of *in vivo* activity against NTHi
Bacteriocin therapy	Haemophilin	Inhibits NTHi growth (Latham et al., 2017), interactions with host cells (Atto et al., 2021a) and prevents colonisation/infection in a mouse model of acute lower respiratory infection (Fulte et al., 2024)	Blocks NTHi access to haem	Efficacy: use of a bacteriocinogenic probiotic may offer more consistent protection due to added bacterial interference for pathogen host cell binding sites Application: requires further investigation in chronic respiratory disease contexts

### 5.1 Antimicrobials that prevent haem/iron assimilation

Several pharmaceutical agents in development target microbial haem-iron acquisition. These agents can be broadly categorised by their intended approach; either those that inhibit haem-iron availability or bacterial acquisition pathways, or toxic haem/iron-mimicking compounds that gain entry to bacterial cells through existing uptake systems (Cassat and Skaar, 2013).

#### 5.1.1 Siderophore synthesis inhibitors

One of the earliest antimicrobials used for tuberculosis treatment, para-aminosalicylic acid, inhibits synthesis of the bacterial siderophore, mycobactin (Brown and Ratledge, 1975). Subsequently, a large number of siderophore biosynthesis inhibitors have been developed that have capacity to prevent bacterial growth under iron-limiting conditions (Ribeiro and Simões, 2019). Flucytosine, a synthetic fluorinated pyrimidine used to treat fungal respiratory tract infections, has also been shown to inhibit expression of the pyoverdine siderophore-biosynthesis gene in *Pseudomonas aeruginosa*, and subsequently suppress pathogenicity in a mouse model of pulmonary infection (Imperi et al., 2013). Similarly, disruption of the function of the pyoverdine protein also mitigated *P. aeruginosa* pathogenesis in a nematode host (Kirienko et al., 2019). Although NTHi possess the capacity to utilise siderophores, genes associated with siderophore biosynthesis have not been identified (Holden and Bachman, 2015). Therefore, the utility of these compounds in restricting NTHi access to iron is limited and may only disrupt a minor iron source by proxy of decreased xenosiderophore production by local microbial communities. Additionally, local microbiota may have the potential to develop adaptive resistance through production of structurally modified siderophores (Abergel et al., 2006; Raffatellu et al., 2009; Holden and Bachman, 2015).

#### 5.1.2 Sideromycins

An alternative approach is the use of xenosiderophores covalently linked to antibiotics, known as sideromycins, that encourage antibiotic uptake through existing iron uptake systems. Sideromycins have demonstrated bactericidal activity against β-lactamase producing Enterobacterales, and other antibiotic-resistant strains of *P. aeruginosa, Stenotrophomonas maltophilia* and *Acinetobacter baumannii*, including producers of the class B metallo-β-lactamases and class C serine-β-lactamases (Page et al., 2010; Brown et al., 2013; Murphy-Benenato et al., 2015). Sideromycins have also been used *in vivo* to successfully treat *P. aeruginosa* infections and prevent systemic infection with *S. pneumoniae* and *Y. enterocolitica* in mouse models (Pramanik et al., 2007; McPherson et al., 2012). However, translation into human use has historically faced diminished clinical utility due to compound instability *in vivo*, emergence of adaptive resistance during exposure, or side-effects accompanying treatment (Ji and Miller, 2012; Page, 2013). As a result, only the novel catechol siderophore-conjugated cephalosporin antibiotic, cefiderocol has progressed beyond the first phase of human safety trials, owing to a unique combination of structural features derived from cefepime and ceftazidime that overcomes that stability problems associated with earlier iterations (Page et al., 2010).

Cefiderocol has recently been approved as a last-line treatment for complicated urinary tract infections and pneumonia caused by antibiotic resistant Gram-negative bacteria, such as carbapenem-resistant *P. aeruginosa, A. baumannii, and K. pneumoniae* (Matsumoto et al., 2017; Ito et al., 2018). Recently, cefiderocol was used to successfully treat ventilator-associated pneumonia caused by *Stenotrophomonas maltophilia* in a preterm neonate (Koirala et al., 2023). However, clinical studies have reported a higher all-cause mortality among patients treated with cefiderocol for hospital-acquired pneumonia, and thus treatment regimens require close monitoring to avoid toxic side-effects, particularly among elderly patients (El-Lababidi and Rizk, 2020). The uncertain safety profile among elderly patients and a lack of data from children lends poorly to routine treatment of NTHi infections which primarily affects these age groups. Additionally, NTHi is adept at scavenging iron from a variety of sources *in vivo* and siderophore-mediated iron acquisition is a minor contributor to total iron acquisition in NTHi (Morton et al., 2010b). This characteristic has been implicated in the intrinsic resistance of NTHi to siderophore-targeting compounds observed *in vivo.* In a nasal colonisation model, production of lipocalin, an acute-phase inhibitor of siderophore-mediated iron uptake, was upregulated but did not affect the ability of *H. influenzae* to acquire host-derived sources of haem-iron (Nelson et al., 2005; Cassat and Skaar, 2013; Leal et al., 2013). Additionally, NTHi cannot utilise catechol-containing siderophores (Williams et al., 1990; Page, 2019) and so is unlikely to possess the necessary machinery to uptake cefiderocol. Thus, it is unlikely that the activity of catechol sideromycins against NTHi will be greater than that of the antibiotic alone. Additionally, production of multiple β-lactamases has been shown to contribute to the emergence of cefiderocol non-susceptibility in several Gram-negative isolates (Kohira et al., 2020). Thus, sideromycins may be vulnerable to the same antibiotic resistance mechanisms faced by traditional antibiotic therapies.

#### 5.1.3 Chelating agents

An alternative approach that mitigates the potential for adaptive resistance involves targeting multiple iron sources through chelating agents (Kontoghiorghes et al., 2010). Chelation therapy prevents accumulation of excess iron and reduces its availability to invading pathogens (Mobarra et al., 2016). This approach has been effective in protecting mice against *K. pneumoniae* pneumonia and dissemination (Michels et al., 2017). However, as is the case with xenosiderophores, iron-containing chelating molecules such as desferrioxamine B (DFO), used in the treatment of iron-loading disorders, can be utilised as an iron source by bacteria that harbour the necessary receptor, including NTHi (Williams et al., 1990; Choon-Mee et al., 2007; Arifin et al., 2014). Chelation therapy with DFO may therefore increase iron availability and increase the risk of infection. More severe infections, and higher liver and kidney bacterial burdens have been demonstrated in DFO-treated mice following intravenous challenge with *S. aureus* (Arifin et al., 2014). A positive correlation between DFO use and higher rates of infection with *S. aureus* and other opportunistic bacteria in patients suffering from thalassemia-associated iron overload has also been reported (Rahav et al., 2006; Lal et al., 2013). Research has therefore moved towards synthetic iron chelators that can be taken orally, such as Deferiprone (DFP) which does not promote bacterial growth (Kim and Shin, 2009). DFP has been found to not only reduce the growth of some strains of common Gram-negative nosocomial pathogens, but also reduce the minimum inhibitory concentrations of antibiotics (Thompson et al., 2012). However, the activity of these compounds against NTHi have not been tested and their effectiveness may be subverted by acquisition of haem-iron.

The majority of NTHi iron requirement is fulfilled by acquisition of haem therefore compounds that restrict haem-assimilation may have higher therapeutic value than those solely targeting iron acquisition. Haem assimilation was first targeted with the analogue gallium-protoporphyrin IX (GaPP), a bactericidal metalloporphyrin that uses existing haem uptake machinery to gain entry to the bacterial cell (Stojiljkovic et al., 1999). Incorporation of Ga(III) in place of iron disrupts the iron-dependent redox process as Ga(III) cannot be reduced to Ga(II) under physiological conditions, and thus cannot be liberated from porphyrins by haem oxygenase (Choi et al., 2019). GaPP has demonstrated inhibitory effects against *A. baumanii* and *P. aeruginosa* in model respiratory cell lines, and other multidrug-resistant Gram-positive and Gram-negative species *in vitro* (Arivett et al., 2015; Hijazi et al., 2017; Hijazi et al., 2018). This compound is also effective against biofilm and intracellular communities (Choi et al., 2019). The activity of GaPP is enhanced when combined with DFP which has been demonstrated by a topical hydrogel with anti-biofilm and antibiotic-potentiating properties against *S. aureus* in an artificial wound model (Richter et al., 2017a; Richter et al., 2017b). Although GaPP has recently been shown to inhibit the growth and intracellular viability of COPD NTHi isolates in limited haemin conditions (Baker et al., 2022), it is limited to topical applications in its current form and cannot be used to prevent future infections. Beyond GaPP, exploration of haem-targeting compounds is scarce. Additional haem-oxygenase inhibitors against *P. aeruginosa* and *Neisseria meningitidis* have only been identified through virtual screening and an *in vitro* growth assay, which were capable of blocking the oxidation of haem at concentrations in excess of that available to pathogens in the respiratory tract (Furci et al., 2007).

### 5.2 Immunotherapies

Recently, vaccine strategies have exploited the haem-dependant NTHi pathogenic mechanisms by incorporating the surface haemoprotein receptor, PE as a vaccine antigen. As previously discussed, PE is an adhesin of NTHi with functions involved in haem binding, storage and inter-bacterial donation (Duell et al., 2016). PE is expressed by 98% of NTHi strains, the epithelial cell-binding region of which is highly conserved among strains (Singh et al., 2010). Serum from mice immunised with recombinant truncated PE demonstrated a strong bactericidal effect against NTHi (Behrouzi et al., 2017). Incorporation of PE in a fusion protein with PilA enhanced immunogenicity and protected against NTHi colonisation and disrupted biofilm integrity in the mouse nasopharynx (Ysebaert et al., 2019). More recently, the PE-PilA fusion protein, combined with Protein D, has completed phase 2 clinical trials, demonstrating an acceptable reactogenicity and safety profile in adults with moderate/severe COPD (Leroux-Roels et al., 2016). Despite these promising results, the isolation of *H. influenzae* from sputum samples did not differ between the vaccine and the placebo group (Wilkinson et al., 2019).

The effectiveness of PE-based approaches may be undermined by the same genetic heterogeneity and phase-variable expression of other potential NTHi surface antigens (Murphy, 2015; Jalalvand and Riesbeck, 2018; Novotny et al., 2019). A high degree of polymorphisms within the gene encoding an NTHi haemoglobin-binding protein has been reported, which alters the protein affinity for iron capture/usage (Duell et al., 2016). This limitation is exacerbated by the high level of redundancy and multi-functionality of NTHi proteins, particularly those involved in the acquisition of haem-iron (Whitby et al., 2009). Therefore, only an antibody response capable of blocking a variety of epitopes may cause sufficient malnutrition to inhibit survival and host-pathogen interactions (Hare, 2017). This approach has been used against uropathogenic *E. coli* strains, where mucosal immunisation with six outer membrane iron receptors or siderophores protected against urinary tract infection in mice (Alteri et al., 2009; Mike et al., 2016). Antibodies targeting the IsdA and IsdB haem-acquisition systems of *S. aureus* protected mice against intravenous challenge (Kim et al., 2010). In addition to targeting iron acquisition proteins, some efficacy has been achieved with vaccines targeting iron homeostasis in pathogens. The Na-APR-1 protease from human hookworm, *Necator americanus*, is essential for enzymatic activity to support blood feeding. Vaccination with a mutated form of Na-APR-1 significantly reduced parasite burden in experimentally-infected canines (Cassat and Skaar, 2013). A similar strategy that targets multiple haem-iron acquisition systems, or their regulation may offer similar protection against NTHi infection. Indeed, interference of multiple NTHi haem-utilisation systems or disruption of the master haem-regulon *fur* has proven effective in attenuating NTHi disease severity/duration in animal models of OM and lung infection (Morton et al., 2004; Morton et al., 2009; Harrison et al., 2013; Szelestey et al., 2013; Rodríguez-Arce et al., 2019).

### 5.3 Bacteriotherapies

The issues inherent to pharmacological- and immunological-based approaches has necessitated the exploration of alternative therapies against NTHi infections. The vital role of haem-iron in the survival of NTHi and other bacteria in the upper respiratory tract raises the stakes for evolutionary conflicts to arise in the struggle for this limiting nutrient (Barber and Elde, 2015). Thus, a commensal bacterium that can outcompete NTHi for haem-iron may have potential as a probiotic therapy by making the environment inhospitable for NTHi growth (De Boeck et al., 2021). A probiotic is defined as a live microorganism that, when administered in adequate amounts, confers a health benefit to the host (Martín and Langella, 2019). Probiotics that outcompete pathogens for iron have demonstrated high levels of protection against infection in the gastrointestinal tract. The Nissle 1917 strain of *E. coli* has been applied as a probiotic treatment that supresses gastroenteritis by outcompeting for the siderophore-mediated iron assimilation of *Salmonella enterica* serovar Typhimurium (Deriu et al., 2013; Holden and Bachman, 2015). This inter-bacterial relationship exposes the protective potential of beneficial microbes to combat pathogens through iron sequestration.

Probiotics administered directly to the upper respiratory tract have close proximity to pathobionts and may therefore interfere with colonisation and development of disease by competing for host cell binding sites and nutrients (e.g. iron) (Falagas et al., 2008). This competitive inhibition appears to be highly effective in formulations where the antagonising commensal bacterium belongs to the same family and occupies the same niche as the pathogenic species. Nasal and oral probiotic sprays containing α-haemolytic streptococcal strains are effective in treating (Manti et al., 2020) and preventing episodes of acute pharyngotonsillitis caused by β-haemolytic group A streptococci, pneumococcal OM in children (Marchisio et al., 2015; Andaloro et al., 2019) and pneumococcal pneumonia in mice (Shekhar et al., 2019). Similarly, intranasal delivery of the closely related commensal *Neisseria lactamica* prevented meningococcal meningitis in mice (Li et al., 2006) and nasal delivery of a commensal *Pasteurellaceae* species was able to delay onset of OM in mice (Granland et al., 2020). Recently, a nasopharyngeal *H. haemolyticus* isolate was discovered that has potent inhibitory activity against NTHi and Hib isolates by outcompeting them for haem (Latham et al., 2017). This ability was attributed to the discovery of a novel haemophore-like protein (now named haemophilin; Hpl) produced by *H. haemolyticus* (Latham et al., 2020). Hpl-producing *H. haemolyticus* strains were shown to outcompete NTHi in a broth co-culture system (Atto et al., 2020) and treatments containing Hpl-producing *H. haemolyticus* (or Hpl alone) protected model respiratory cell lines from NTHi attachment and invasion (Atto et al., 2021a). Furthermore, a small-scale epidemiological study of 257 healthy adults in Australia found a strong correlation between pharyngeal carriage of *H. haemolyticus* strains containing *hpl* and a reduced likelihood and density of NTHi co-colonisation, compared to participants colonised with *H. haemolyticus* strains incapable of producing Hpl (Atto et al., 2021b). Recently, intranasal administration of Hpl or Hpl-producing *H. haemolyticus* reduced respiratory tract colonisation and infection with NTHi in a mouse model of acute lower airway infection (Fulte et al., 2024). Hpl may therefore have potent clinical utility in preventing NTHi infections; however, further studies are needed to assess efficacy in chronic respiratory disease contexts. Probiotic approaches (such as Hpl) that exploit NTHi haem-iron dependency deliver several benefits over standard antibiotic therapy which make them an asset to dampening emergent resistance. For example, their narrow spectrum of activity does not provoke collateral effects on the whole microbiota or promote enrichment of resistant clones/strains (Rea et al., 2010; Hols et al., 2019). Additionally, the presence of a competitive probiotic in the nasopharynx may also provide additional inhibition of pathogen colonisation, or prevent strain replacement through interference for host cell binding sites (Drutz et al., 1966).

## 6 Conclusion

The growing prevalence of resistance to first- and second-line antibiotics, in the absence of an effective preventative strategy necessitates the need for alternative therapies that can reduce the enormous disease burden associated with NTHi infections. Strategies that target haem-iron assimilation of NTHi have a potentially high impact on the ability of NTHi to survive and cause disease within host airways. Unlike pharmacological- and immunological-based approaches, bacteriotherapy may provide an effective strategy that both treats, and prevents NTHi infections, which is not compromised by antigenic heterogeneity or bacterial resistance mechanisms. However, targeting the haem-iron assimilation of NTHi requires careful consideration and improved modelling of haem-iron sources and fluctuations present in respiratory niches during health and disease.
